# Reasons and Factors Contributing to Chinese Patients’ Preference for Ustekinumab in Crohn’s Disease: A Multicenter Cross-Sectional Study

**DOI:** 10.3389/fphar.2021.736149

**Published:** 2021-11-22

**Authors:** Lingya Yao, Xiao Zhu, Bule Shao, Rongbei Liu, Zhilun Li, Lexi Wu, Jin Chen, Qian Cao

**Affiliations:** ^1^ Department of Gastroenterology, Sir Run Run Shaw Hospital, College of Medicine Zhejiang University, Hangzhou, China; ^2^ Medical Affairs, Janssen, China

**Keywords:** Crohn’s disease, ustekinumab (UST), preference, Chinese patients, multicenter cross-sectional study

## Abstract

**Background and Aims:** Ustekinumab (UST) was approved in China for treating moderate-to-severe Crohn’s disease (CD) in 2020. We aimed to identify the reasons and possible contributing factors for UST preference in Chinese patients with CD.

**Methods:** We conducted a multicenter cross-sectional survey among patients with moderate to severe CD who underwent UST treatment in 27 hospitals. Patients completed a 46-item questionnaire that included information on demographics, clinical characteristics, reasons in favor of UST and shared decision-making perception. Logistic regression analysis was performed to examine the predictive factors of different UST preferences.

**Results:** Overall, 127 patients (73 males; mean age, 25.9 ± 9.9 years) completed the questionnaire. Most patients (74.8%) had biologic failure. The most common reason for the latest treatment disconnection was unresponsiveness to the previous medications. The major UST information sources were physicians (96.1%). Nearly half of the patients (44.9%) reported shared decision making regarding UST treatment. No difference was found in the decision-making patterns in terms of sex and age. The most influential reason for UST preference was “effectiveness” (77%, 98/127), followed by “safety” (65%, 83/127), “frequency of administration” (39%, 49/127), and “mode of administration” (37%, 47/127). Multivariate logistic regression analysis revealed that a positive self-rated health status was a contributing factor for UST preference with a low frequency of administration.

**Conclusion:** This is the first multicenter survey of Chinese patients with CD to identify the possible contributing factors for UST preference. Treatment choice should be discussed with patients because individual preferences are determined by diverse factors.

## Introduction

Crohn’s disease (CD) is a chronic inflammatory disorder of the gastrointestinal tract characterized by a perpetuated mucosal immune response, often requiring life-long medical treatment (Vermeire et al., 2007). Although CD has been considered remarkably rare in Asian countries compared with Western countries, its incidence and prevalence have been increasing recently in China (Qiao and Ran, 2019). The exact etiology of the disease has not been fully clarified; interleukin-23 (IL-23) is regarded as one of the main pathophysiological components involved in the retractable mucosal inflammation of the gut (Petagna et al., 2020).

Therapeutic medications for patients with CD, which have been endorsed by the National Medical Products Administration of China, include glucocorticoids, immunosuppressants, tumor necrosis factor (TNF) antagonists, and integrin inhibitors (Biancone et al., 2003; Colombel et al., 2007; Sandborn et al., 2012). Recently, several anti-IL23 agents, including ustekinumab (UST), guselkumab, tildrakizumab, and risankizumab, have been administered as new therapeutic strategies for achieving sustained clinical remission and mucosal healing in Western populations with CD (Ma et al., 2019; Almradi et al., 2020). However, only UST has been approved in China for intravenous induction therapy of moderate-to-severe CD since 2017 as a fully human monoclonal antibody to IL-12/23p40. Since 2020, UST has been utilized among the Chinese population with CD. UST can be administered by subcutaneous or intravenous injection, the latter of which is the only usage for infliximab (IFX), the most common biological agent for CD treatment in China.

The decision to use UST is based on many factors, including information sources and perceptions about UST. Rigorous patient involvement in decision making plays a vital role in the management of CD because patients, who are actively involved in decision making regarding their treatment, have a greater possibility of acquiring satisfaction and better clinical outcomes (van den Bemt and van Lankveld, 2007; Siegel, 2012). Therefore, it is essential for patients to be actively involved in the decision-making process (Baars et al., 2010). Moreover, a recent study demonstrated that patients with psoriasis living in the Netherlands preferred UST, mainly due to its therapeutic effects (van Muijen et al., 2021). However, no study has been conducted to investigate the reasons and factors associated with UST preference in patients with CD in the Chinese population.

Therefore, we aimed to identify the reasons and possible contributing factors for UST preference in the profiles of Chinese patients. In addition, we also sought to investigate the information sources and decision-making patterns of the Chinese population with CD.

## Materials and methods

### Study design and population

We conducted a multicenter cross-sectional survey among patients with moderate-to-severe CD who underwent UST treatment. Twenty-seven hospitals in China participated in this study from October 2020 to February 2021, and patients were enrolled consecutively. Male and female patients enrolled in the study had a diagnosis established by a combination of clinical symptoms, endoscopic examination, pathologic examination, and the absence of alternative diagnoses (Lichtenstein et al., 2018). CD activity was classified based on the Crohn’s disease activity index (Conigliaro et al., 2021). The studies involving human participants were reviewed and approved by the China Ethics Committee of Registering Clinical Trials. The patients/participants provided their written informed consent to participate in this study.

### Questionnaires

“The Questionnaire in Chinese patients with moderate to severe CD who underwent UST treatment” was done just after the decision of treatment with ustekinumab. It consisted of 46 items, which could be completed by the patients within 10 min. First, the patients were asked 17 questions concerning their demographic information, including the age, body mass index, and the history of alcohol intake. Second, 25 questions about clinical characteristics were asked, including the date of onset, date of diagnosis, history of perianal surgery, and previous medications. Finally, the remaining four questions pertained to the reasons for UST preference, information sources for UST, and shared decision-making perception required answers. The detailed contents of the questionnaire are shown in Supplementary Table S1.

### Treatment strategy

Patients received different doses of UST according to their body weight: 1) week 0: UST intravenous injection of 260 mg when weight was ≤55 kg, 390 mg when weight was >55 kg, but ≤85 kg and 520 mg when weight was >85 kg; 2) week 8: UST subcutaneous injection of 90 mg; and 3) every 12 weeks after week 8: subcutaneous or intravenous injection of the same dose of UST, dependent upon the physicians in different centers.

### Statistical analysis

Continuous variables are presented as mean ± standard deviation or median (quartiles, Q1–Q3), and categorical variables are presented as frequencies and percentages. Different sources of information on UST and strategy for UST drug selection were compared between sexes and ages using the Chi-square test. Student’s t-test or the Wilcoxon signed-rank test was used for continuous variables, while the Chi-square test or Fisher’s exact test was used for categorical variables to compare the characteristics between groups (groups with different preferences toward the frequency of UST administration, different preferences toward modes of UST administration, and different preferences toward impacts of UST treatment on daily life). Variables with a *p*-value ≤0.1 were selected to fit a multivariate logistic model to explore potentially influential factors that led to different reasons for choosing UST. All tests were two sided, and the results were considered statistically significant at a *p*-value <0.05. All analyses were conducted using SAS 9.4 (SAS Institute, Cary NC, USA), and the graphs were plotted using R software (version 4.1) (R. R Development Core Team, 2021) with the ggplot2 package. (Wickham, 2016)

## Results

### Baseline characteristics

A total of 127 patients diagnosed with CD were enrolled, and 73 (57.5%) of them were males. The mean age at survey was 31.0 ± 11.3 years, the mean age at diagnosis was 25.9 ± 9.9 years, and the median interval between onset and diagnosis was 9.4 (1.8–48.2) months. Approximately half of the patients (48.8%) had a university education background, and 59 (46.5%) household income per capita of the patients were less than 5,000 yuan/month. Forty (31.5%) and 61 (48.0%) patients had good and fair health statuses, respectively. L1 (ileal) and B2 (stricturing) were the most common location and disease behavior of CD. Majority of the patients (74.8%) had biologic failure (mainly TNF inhibitors); the most common reason for the latest treatment discontinuation was unresponsiveness to the previous medications. Other demographic and clinical characteristics of the participants are presented in Table 1.

**TABLE 1:** Basic characteristics of participants (*n* = 127).

Variable	*n* (%)/Mean ± SD
Age at survey, year	31.0 ± 11.3
Age at diagnosis, year	25.9 ± 9.9
Interval between onset and diagnosis, month [median (Q1–Q3)]	9.4 (1.8–48.2)
BMI, kg/m^2^ ^a^	19.5 ± 4.2
Male	73 (57.5%)
Smoking	
Current smoker	8 (6.3%)
Non-smoker	112 (88.2%)
Quit	7 (5.5%)
Alcohol consumption	
<1 time/month	18 (14.2%)
1–4 times/month	4 (3.1%)
Never	105 (82.7%)
Education attainment	
High school or under	29 (22.8%)
College	25 (19.7%)
University	62 (48.8%)
Graduate or above	11 (8.7%)
Marital status	
Single	68 (53.5%)
Married	56 (44.1%)
Divorced	3 (2.4%)
Employment status	
Unemployed	52 (40.9%)
Employed	52 (40.9%)
Other	23 (18.2%)
Household income per capita (Yuan/month)	
≤5,000	59 (46.5%)
5,001–10,000	46 (36.2%)
10,001–20,000	17 (13.4%)
≥20,001	5 (3.9%)
Health insurance	
Yes	111 (87.4%)
No	7 (5.5%)
Other	9 (7.1%)
Self-rated health	
Very good	4 (3.1%)
Good	40 (31.5%)
Fair	61 (48.0%)
Poor	22 (17.3%)
Disease location	
Ileal [L1]	47 (37.0%)
Colonic [L2]	34 (26.8%)
Ileocolonic [L3]	25 (19.7%)
Upper gastrointestinal disease [L4]	3 (2.4%)
L1 + L4	1 (0.8%)
L2 + L4	2 (1.6%)
L3 + L4	8 (6.3%)
Unknown	7 (5.5%)
Montreal classification	
Nonstricturing, nonpenetrating [B1]	19 (15.0%)
Stricturing [B2]	44 (34.6%)
Penetrating [B3]	6 (4.7%)
Perianal involvement [B1p/B2p/B3p]	42 (33.1%)
Unknown	16 (12.6%)
Family history	
No	125 (98.4%)
Yes	2 (1.6%)
History of intestinal surgery	
No	85 (66.9%)
Yes	42 (33.1%)
History of perianal surgery	
No	72 (56.7%)
Yes	55 (43.3%)
History of hormone therapy	
Yes	40 (31.5%)
No	79 (62.2%)
Not sure	8 (6.3%)
History of immunosuppressant therapy	
Yes	66 (52.0%)
No	55 (43.3%)
Not sure	6 (4.7%)
History of biologic agent therapy	
No	28 (22.0%)
Yes	99 (78.0%)
Reason of recent drug withdrawal^b^	
Unresponsive	71 (55.9%)
Intolerant	14 (11.0%)
Adverse effect	13 (10.2%)
Economic reason	17 (13.4%)
Other	34 (26.8%)

^a^ *n* = 112.

b Multiple choice.

**FIGURE 1:**
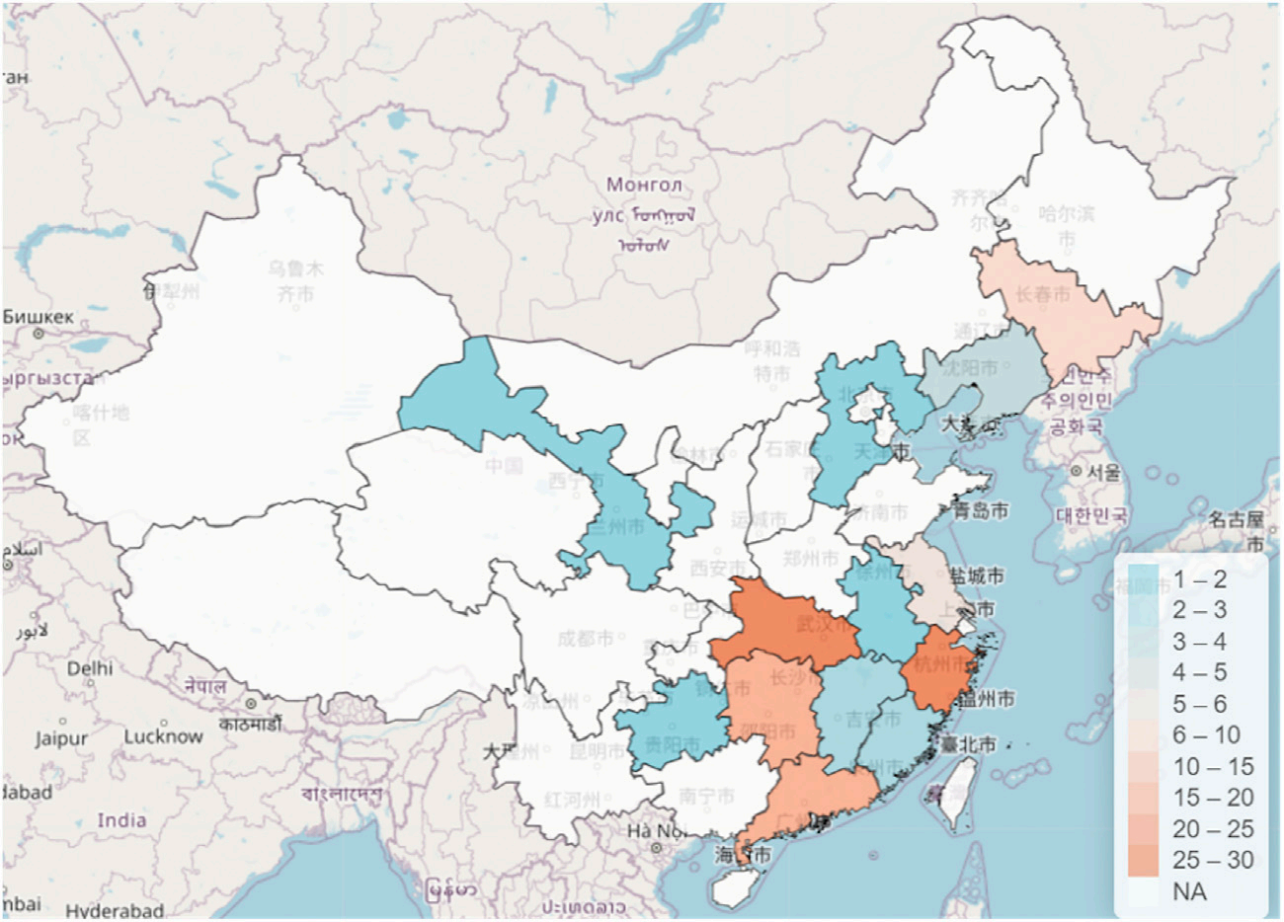
Distribution of participants.

As shown in Figure 1, the participants were mostly from the Zhejiang and Hubei Provinces, followed by the Guangdong and Hunan Provinces. Furthermore, there were participants from Fujian, Jiangxi, Anhui, Guizhou, Qinghai, and Hebei Provinces.

### Source of information on ustekinumab

As shown in Table 2, the major information source of UST treatment for CD was from physicians (122, 96.1%), followed by family/friends/ associations of patients (21, 16.5%), internet (13, 10.2%), and books or television (5,3.9%). Except for mere sex differences for source from physicians (100% versus 93.2%; *p* = 0.0497), no significant difference was observed among other information sources. In addition, a similar pattern was found between different age subgroups at diagnosis when comparing the sources of information.

**TABLE 2:** Comparison of source of information on ustekinumab (UST).

Source of information	All	Sex		*p*-Value	Age at survey		*p*-Value
	*n* (%)	Female *n* (%)	Male *n* (%)		<30 *n* (%)	≥30 *n* (%)	
Physicians	122 (96.1%)	54 (100.0%)	68 (93.2%)	**0.0497**	71 (95.9%)	51 (96.2%)	0.9361
Family/friends/association of patients	21 (16.5%)	8 (14.8%)	13 (17.8%)	0.6535	12 (16.2%)	9 (17.0%)	0.9089
Internet	13 (10.2%)	3 (5.6%)	10 (13.7%)	0.1345	9 (12.2%)	4 (7.5%)	0.3975
Books/TV	5 (3.9%)	2 (3.7%)	3 (4.1%)	0.9074	4 (5.4%)	1 (1.9%)	0.3147
Others	3 (2.4%)	0 (0.0%)	3 (4.1%)	0.1317	2 (2.7%)	1 (1.9%)	0.7653

Bold value shows that when comparing source of information on UST, male patients were more likely to acquire information from physicians than women.

### Strategy for ustekinumab drug choice

Table 3 displays the choice strategy for UST among the different sex and age subgroups. Nearly half of the patients (57, 44.9%) reported a shared decision making regarding UST treatment, followed by 49 (38.6%), self-decision with explanations from physicians. No differences were found in the decision-making patterns in terms of sex and age. Considering the pandemic situation (COVID) during the study period, we further analyzed whether there were differences between patients from Hubei Province and other provinces in the aspect of strategy for UST drug choice. As shown in Supplementary Table S2, no significant difference was found in strategies on ustekinumab drug choice between patients from Hubei Province and other provinces.

**TABLE 3:** Comparison of strategy on UST drug choice.

Strategy on drug choose	All	Sex		*p*-Value	Age at survey		*p*-Value
	*n* (%)	Female *n* (%)	Male *n* (%)		<30 *n* (%)	≥30 *n* (%)	
Decided by physicians and me	57 (44.9%)	23 (42.6%)	34 (46.6%)	0.6055	35 (47.3%)	22 (41.5%)	0.6405
Decided by myself after explanations from physicians	49 (38.6%)	20 (37.0%)	29 (39.7%)		26 (35.1%)	23 (43.4%)	
Decided by physicians	21 (16.5%)	11 (20.4%)	10 (13.7%)		13 (17.6%)	8 (15.1%)	

### Reasons for ustekinumab preference

**FIGURE 2:**
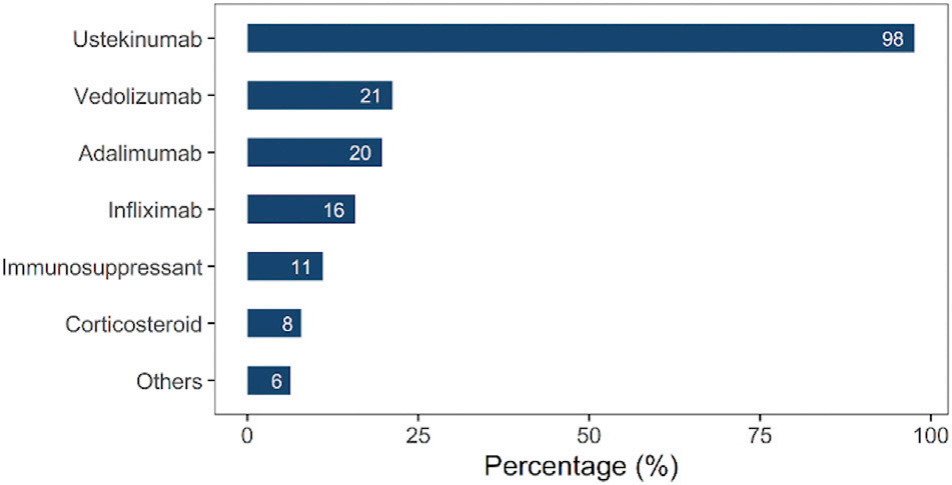
Candidate drugs before ustekinumab (UST) therapy.

As shown in Figure 2, UST (98%) was the major candidate option for CD treatment. Other candidate drugs included vedolizumab (21%), adalimumab (20%), IFX (16%), immunosuppressants (11%), and corticosteroids (8%).

**FIGURE 3:**
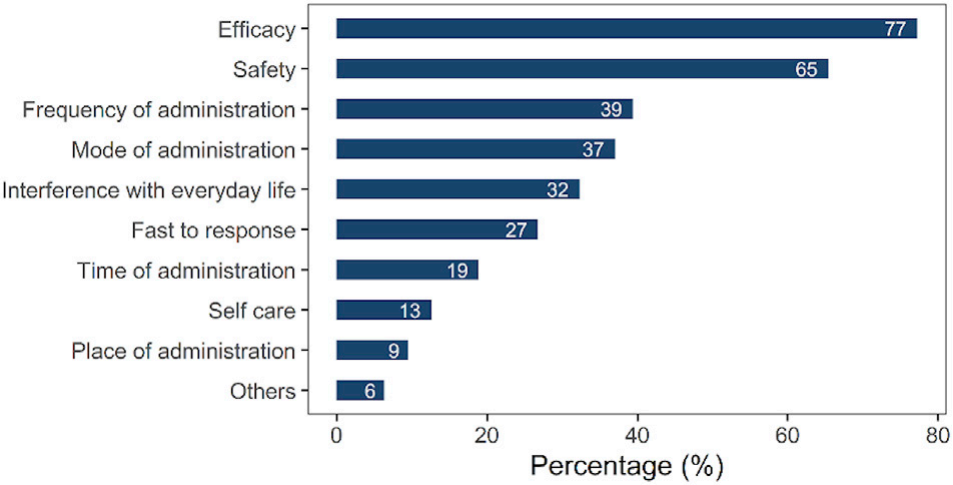
Reasons for the choice of UST.

To investigate the various reasons for choosing UST as treatment medication among the Chinese population, we designed multiple choice answers not only in terms of efficacy and safety but also in terms of frequency of administration, mode of administration, interference with everyday life, fast to respond, and time of administration, which had been scarcely detected in previous studies. Efficacy (77%) and safety (65%), frequency of administration (39%), mode of administration (intravenous or subcutaneous) (37%), and decreased interference with everyday life (32%) were the most common options for choosing UST. Unusual reasons included “fast to respond” (27%), “time of administration” (19%), “self-care” (13%), and “place of administration” (9%), as shown in Figure 3. In addition, we also analyzed whether there were differences between patients from Hubei Province and other provinces in reasons for UST preference. As shown in Supplementary Table S3, despite a slightly higher rate of frequency of administration observed in patients from Hubei Province (*p* = 0.007), no significant difference was found in other reasons for UST preference between patients from Hubei Province and other provinces.

### Factors contributing to preference for ustekinumab

Next, we investigated the possible demographic characteristics contributing to the preference for UST with frequency of administration, mode of administration, and interference with everyday life, separately.

In the first subgroup analysis, patients were divided according to different preferences regarding the frequency of UST administration. Table 4 shows that self-rate health was significantly different (*p* = 0.013) among patients who chose UST for treatment, regardless of the reason for low-frequency administration. Other factors found to be associated with the UST preference, with a low frequency of administration in the univariate analysis, were the age at diagnosis (*p* = 0.091) and employment status (*p* = 0.105). In the multivariate analysis, a positive self-rated health status (fair, *p* = 0.044; good, *p* = 0.004; and very good, *p* = 0.007) was a contributing factor for UST preference with a low frequency of administration, as shown in Table 5.

**TABLE 4:** Characteristics of participants with different preference toward frequency of UST administration.

Variable	Prefer low frequency of UST administration		*p*-Value
	No (*n* = 77)	Yes (*n* = 50)	
Age at diagnosis, year	27.1 ± 10.2	24.0 ± 9.1	0.091
Interval between onset and diagnosis, month [median (Q1–Q3)]	5.08 (1.17–48.3)	12.9 (3.08–42.9)	0.229^#^
BMI, kg/m^2^ ^a^	19.7 ± 4.9	19.3 ± 2.8	0.552
Male	31 (40.3%)	23 (46.0%)	0.523
Smoking			0.702*
Current smoker	4 (5.2%)	4 (8.0%)	—
Nonsmoker	68 (88.3%)	44 (88.0%)	—
Quit	5 (6.5%)	2 (4.0%)	—
Alcohol consumption			1.000*
<1 time/month	11 (14.3%)	7 (14.0%)	—
1–4 times/month	3 (3.9%)	1 (2.0%)	—
Never	63 (81.8%)	42 (84.0%)	—
Education attainment			0.492
High school or under	14 (18.2%)	15 (30.0%)	—
College	16 (20.8%)	9 (18.0%)	—
University	40 (51.9%)	22 (44.0%)	—
Graduate or above	7 (9.1%)	4 (8.0%)	—
Marital status			0.147*
Single	36 (46.8%)	32 (64.0%)	—
Married	39 (50.6%)	17 (34.0%)	—
Divorced	2 (2.6%)	1 (2.0%)	—
Employment status			0.105
Unemployed	29 (37.7%)	23 (46.0%)	—
Employed	37 (48.1%)	15 (30.0%)	—
Other	11 (14.3%)	12 (24.0%)	—
Household income per capita (Yuan/month)			0.755*
≤5,000	37 (48.1%)	22 (44.0%)	—
5,001–10,000	27 (35.1%)	19 (38.0%)	—
10,001–20,000	11 (14.3%)	6 (12.0%)	—
≥20,001	2 (2.6%)	3 (6.0%)	—
Health insurance			0.654*
Yes	66 (85.7%)	45 (90.0%)	—
No	4 (5.2%)	3 (6.0%)	—
Other	7 (9.1%)	2 (4.0%)	—
Self-rated health			**0.013**
Very good	2 (2.6%)	2 (4.0%)	—
Good	20 (26.0%)	20 (40.0%)	—
Fair	35 (45.5%)	26 (52.0%)	—
Poor	20 (26.0%)	2 (4.0%)	—
Disease location			0.765*
Ileal [L1]	30 (39.0%)	17 (34.0%)	—
Colonic [L2]	23 (29.9%)	11 (22.0%)	—
Ileocolonic [L3]	14 (18.2%)	11 (22.0%)	—
Upper gastrointestinal disease [L4]	1 (1.3%)	2 (4.0%)	—
L1 + L4	1 (1.3%)	0 (0.0%)	—
L2 + L4	1 (1.3%)	1 (2.0%)	—
L3 + L4	4 (5.2%)	4 (8.0%)	—
Unknown	3 (3.9%)	4 (8.0%)	—
Montreal classification			0.740
Nonstricturing, nonpenetrating [B1]	12 (15.6%)	7 (14.0%)	—
Stricturing [B2]	26 (33.8%)	18 (36.0%)	—
Penetrating [B3]	3 (3.9%)	3 (6.0%)	—
Perianal involvement [B1p/B2p/B3p]	24 (31.2%)	18 (36.0%)	—
Unknown	12 (15.6%)	4 (8.0%)	—
Family history			0.519*
No	75 (97.4%)	50 (100.0%)	—
Yes	2 (2.6%)	0 (0.0%)	—
History of intestinal surgery			0.328
No	49 (63.6%)	36 (72.0%)	—
Yes	28 (36.4%)	14 (28.0%)	—
History of perianal surgery			0.899
No	44 (57.1%)	28 (56.0%)	—
Yes	33 (42.9%)	22 (44.0%)	—
History of hormone therapy			0.318
Yes	27 (35.1%)	13 (26.0%)	—
No	44 (57.1%)	35 (70.0%)	—
Not sure	6 (7.8%)	2 (4.0%)	—
History of immunosuppressant therapy			0.479*
Yes	43 (55.8%)	23 (46.0%)	—
No	30 (39.0%)	25 (50.0%)	—
Not sure	4 (5.2%)	2 (4.0%)	—
History of biologic agent therapy			0.669
No	16 (20.8%)	12 (24.0%)	—
Yes	61 (79.2%)	38 (76.0%)	—
Reason of recent drug withdrawal^b^			
Unresponsive	44 (57.1%)	27 (54.0%)	0.727
Intolerant	9 (11.7%)	5 (10.0%)	0.767
Adverse effect	6 (7.8%)	7 (14.0%)	0.260
Economic reason	9 (11.7%)	8 (16.0%)	0.486
Other	19 (24.7%)	15 (30.0%)	0.508

Bold value shows that patients who chose UST due to low frequency were more likely to have a better self-rated health status.

^a^ *n* = 112.

b Multiple choice.

^#^Wilcoxon test.

*Fisher’s exact test.

**TABLE 5:** Multivariate logistic regression analysis of predictive factors for preference toward low frequency of UST administration.

Variable	Or (95% CI)	p-Value
Male	0.87 (0.39–1.93)	0.733
Age at diagnosis	0.98 (0.92–1.04)	0.531
Employment status		
Unemployed	Ref	—
Employed	0.97 (0.28–3.45)	0.967
Other	2.46 (0.63–9.57)	0.195
Self-rated health		
Poor	Ref	—
Fair	15.21 (1.07–216.10)	**0.044**
Good	13.88 (2.38–80.98)	**0.004**
Very good	10.62 (1.92–58.83)	**0.007**
Education attainment		
High school or under	Ref	—
College	0.34 (0.09–1.31)	0.118
University	0.37 (0.12–1.16)	0.089
Graduate or above	0.51 (0.09–2.87)	0.441

Bold value shows that patients who chose UST due to low frequency were more likely to have a better self-rated health status.

In the second subgroup analysis, patients were divided according to different preferences for the mode of UST administration. History of hormone therapy (*p* = 0.013), history of biologic agent therapy (*p* = 0.040), and other reasons for recent drug withdrawal (*p* = 0.025) showed differences in the univariate analysis. Similar results revealed that self-rate health was a contributing factor for UST preference due to convenient administration both in the univariate analysis (*p* = 0.010) and multivariate analysis (very good, *p* = 0.008), as shown in Tables 6 and 7.

**TABLE 6:** Characteristics of participants with different preference toward mode of UST administration.

Variable	Prefer convenience of UST administration		*p*-Value
	No (*n* = 80)	Yes *n* = 47	
Age at diagnosis, year	26.6 ± 10.3	24.6 ± 9.0	0.281
Interval between onset and diagnosis, month [median (Q1–Q3)]	7.67 (1.79–54.8)	10.3 (1.8–42.9)	0.832^#^
BMI, kg/m^2^ ^a^	19.7 ± 5.1	19.3 ± 2.2	0.592
Male	33 (41.3%)	21 (44.7%)	0.706
Smoking			1.000*
Current smoker	5 (6.3%)	3 (6.4%)	—
Nonsmoker	70 (87.5%)	42 (89.4%)	—
Quit	5 (6.3%)	2 (4.3%)	—
Alcohol consumption			0.196*
<1 time/month	8 (10.0%)	10 (21.3%)	—
1–4 times/month	3 (3.8%)	1 (2.1%)	—
Never	69 (86.3%)	36 (76.6%)	—
Education attainment			0.731
High school or under	18 (22.5%)	11 (23.4%)	—
College	18 (22.5%)	7 (14.9%)	—
University	38 (47.5%)	24 (51.1%)	—
Graduate or above	6 (7.5%)	5 (10.6%)	—
Marital status			0.492*
Single	41 (51.3%)	27 (57.4%)	—
Married	36 (45.0%)	20 (42.6%)	—
Divorced	3 (3.8%)	0 (0.0%)	—
Employment status			0.580
Unemployed	30 (37.5%)	22 (46.8%)	—
Employed	35 (43.8%)	17 (36.2%)	—
Other	15 (18.8%)	8 (17.0%)	—
Household income per capita (Yuan/month)			0.582*
≤5,000	40 (50.0%)	19 (40.4%)	—
5,001–10,000	28 (35.0%)	18 (38.3%)	—
10,001–20,000	10 (12.5%)	7 (14.9%)	—
≥20,001	2 (2.5%)	3 (6.4%)	—
Health insurance			0.162*
Yes	69 (86.3%)	42 (89.4%)	—
No	3 (3.8%)	4 (8.5%)	—
Other	8 (10.0%)	1 (2.1%)	—
Self-rated health			0.010*
Very good	2 (2.5%)	2 (4.3%)	—
Good	25 (31.3%)	15 (31.9%)	—
Fair	33 (41.3%)	28 (59.6%)	—
Poor	20 (25.0%)	2 (4.3%)	—
Disease location			0.611*
Ileal [L1]	30 (37.5%)	17 (36.2%)	—
Colonic [L2]	22 (27.5%)	12 (25.5%)	—
Ileocolonic [L3]	15 (18.8%)	10 (21.3%)	—
Upper gastrointestinal disease [L4]	1 (1.3%)	2 (4.3%)	—
L1 + L4	1 (1.3%)	0 (0.0%)	—
L2 + L4	1 (1.3%)	1 (2.1%)	—
L3 + L4	7 (8.8%)	1 (2.1%)	—
Unknown	3 (3.8%)	4 (8.5%)	—
Montreal classification			0.733
Nonstricturing, nonpenetrating [B1]	11 (13.8%)	8 (17.0%)	—
Stricturing [B2]	29 (36.3%)	15 (31.9%)	—
Penetrating [B3]	3 (3.8%)	3 (6.4%)	—
Perianal involvement [B1p/B2p/B3p]	25 (31.3%)	17 (36.2%)	—
Unknown	12 (15.0%)	4 (8.5%)	—
Family history			0.530*
No	78 (97.5%)	47 (100.0%)	—
Yes	2 (2.5%)	0 (0.0%)	—
History of intestinal surgery			0.166
No	50 (62.5%)	35 (74.5%)	—
Yes	30 (37.5%)	12 (25.5%)	—
History of perianal surgery			0.326
No	48 (60.0%)	24 (51.1%)	—
Yes	32 (40.0%)	23 (48.9%)	—
History of hormone therapy			**0.013**
Yes	32 (40.0%)	8 (17.0%)	—
No	42 (52.5%)	37 (78.7%)	—
Not sure	6 (7.5%)	2 (4.3%)	—
History of immunosuppressant therapy			0.227*
Yes	46 (57.5%)	20 (42.6%)	—
No	30 (37.5%)	25 (53.2%)	—
Not sure	4 (5.0%)	2 (4.3%)	—
History of biologic agent therapy			**0.040**
No	13 (16.3%)	15 (31.9%)	—
Yes	67 (83.8%)	32 (68.1%)	—
Reason of recent drug withdrawal^b^			
Unresponsive	48 (60.0%)	23 (48.9%)	0.225
Intolerant	10 (12.5%)	4 (8.5%)	0.488
Adverse effect	9 (11.3%)	4 (8.5%)	0.766*
Economic reason	11 (13.8%)	6 (12.8%)	0.875
Other	16 (20.0%)	18 (38.3%)	**0.025**

Bold value shows that a history of hormone therapy (*p* = 0.013), history of biologic agent therapy (*p* = 0.040) and other reasons for recent drug withdrawal (*p* = 0.025) showed differences in the univariate analysis. Similar results revealed that self-rate health was a contributing factor for UST preference due to convenient administration both in the univariate analysis (*p* = 0.010) and multivariate analysis (very good, *p* = 0.008).

Note. ^a^ *n* = 112.

b Multiple choice.

^#^Wilcoxon test.

*Fisher’s exact test.

**TABLE 7:** Multivariate logistic regression analysis of predictive factors for preference toward convenience of UST administration.

Variable	Or (95% CI)	*p*-Value
Male	0.81 (0.36–1.83)	0.605
Age at diagnosis	0.98 (0.94–1.03)	0.480
Self-rated health		
Poor	Ref	—
Fair	6.17 (0.48–78.9)	0.162
Good	4.40 (0.83–23.2)	0.081
Very good	8.52 (1.73–42.0)	**0.008**
History of hormone therapy		
No	Ref	—
Yes	0.38 (0.14–1.04)	0.059
Not sure	0.59 (0.10–3.67)	0.572
History of biologic agent therapy	0.60 (0.21–1.72)	0.341
Reason of recent drug withdrawal (others)	1.87 (0.73–4.77)	0.190

Bold value shows that a history of hormone therapy (*p* = 0.013), history of biologic agent therapy (*p* = 0.040) and other reasons for recent drug withdrawal (*p* = 0.025) showed differences in the univariate analysis. Similar results revealed that self-rate health was a contributing factor for UST preference due to convenient administration both in the univariate analysis (*p* = 0.010) and multivariate analysis (very good, *p* = 0.008).

When analyzed separately according to different preferences toward the impact of UST administration on everyday life, self-rate health was still significantly different (*p* = 0.008) between the two groups, regardless of whether they had a low impact on everyday life. Other factors found to be associated with impact preference in the univariate analysis were a history of hormone therapy (*p* = 0.067) and economic reasons for recent drug withdrawal (*p* = 0.050) (Table 8). Furthermore, a fitful multivariate logistic model was selected to explore potential factors that were relevant to the impact preference for choosing UST. In line with this, patients with a positive self-rated health status (good, *p* = 0.020; very good, *p* = 0.008) were correlated with a higher possibility of UST preference with a low interference in everyday life, as shown in Table 9.

**TABLE 8:** Characteristics of participants with different preference toward impact of UST administration on everyday life.

Variable	Prefer low impact of UST administration on everyday life		*p*-Value
	No (*n* = 86)	Yes (*n* = 41)	
Age at diagnosis, year	26.9 ± 10.3	23.6 ± 8.5	0.077
Interval between onset and diagnosis, month [median (Q1–Q3)]	7.67 (1.79–54.8)	10.3 (1.8–42.9)	0.630^#^
BMI, kg/m^2^ ^a^	19.4 ± 4.4	19.9 ± 3.7	0.520
Male	34 (39.5%)	20 (48.8%)	0.324
Smoking			0.369*
Current smoker	4 (4.7%)	4 (9.8%)	—
Nonsmoker	76 (88.4%)	36 (87.8%)	—
Quit	6 (7.0%)	1 (2.4%)	—
Alcohol consumption			1.000*
<1 time/month	12 (14.0%)	6 (14.6%)	—
1–4 times/month	3 (3.5%)	1 (2.4%)	—
Never	71 (82.6%)	34 (82.9%)	—
Education attainment			0.800
High school or under	19 (22.1%)	10 (24.4%)	—
College	19 (22.1%)	6 (14.6%)	—
University	41 (47.7%)	21 (51.2%)	—
Graduate or above	7 (8.1%)	4 (9.8%)	—
Marital status			0.099*
Single	41 (47.7%)	27 (65.9%)	—
Married	43 (50.0%)	13 (31.7%)	—
Divorced	2 (2.3%)	1 (2.4%)	—
Employment status			0.764
Unemployed	35 (40.7%)	17 (41.5%)	—
Employed	34 (39.5%)	18 (43.9%)	—
Other	17 (19.8%)	6 (14.6%)	—
Household income per capita (Yuan/month)			0.059*
≤5,000	42 (48.8%)	17 (41.5%)	—
5,001–10,000	29 (33.7%)	17 (41.5%)	—
10,001–20,000	14 (16.3%)	3 (7.3%)	—
≥20,001	1 (1.2%)	4 (9.8%)	—
Health insurance			0.759*
Yes	75 (87.2%)	36 (87.8%)	—
No	4 (4.7%)	3 (7.3%)	—
Other	7 (8.1%)	2 (4.9%)	—
Self-rated health			0.008*
Very good	3 (3.5%)	1 (2.4%)	—
Good	25 (29.1%)	15 (36.6%)	—
Fair	37 (43.0%)	24 (58.5%)	—
Poor	21 (24.4%)	1 (2.4%)	—
Disease location			0.983*
Ileal [L1]	33 (38.4%)	14 (34.1%)	—
Colonic [L2]	22 (25.6%)	12 (29.3%)	—
Ileocolonic [L3]	17 (19.8%)	8 (19.5%)	—
Upper gastrointestinal disease [L4]	2 (2.3%)	1 (2.4%)	—
L1 + L4	1 (1.2%)	0 (0.0%)	—
L2 + L4	1 (1.2%)	1 (2.4%)	—
L3 + L4	6 (7.0%)	2 (4.9%)	—
Unknown	4 (4.7%)	3 (7.3%)	—
Montreal classification			0.237
Nonstricturing, nonpenetrating [B1]	15 (17.4%)	4 (9.8%)	—
Stricturing [B2]	31 (36.0%)	13 (31.7%)	—
Penetrating [B3]	2 (2.3%)	4 (9.8%)	—
Perianal involvement [B1p/B2p/B3p]	26 (30.2%)	16 (39.0%)	—
Unknown	12 (14.0%)	4 (9.8%)	—
Family history			1.000*
No	84 (97.7%)	41 (100.0%)	—
Yes	2 (2.3%)	0 (0.0%)	—
History of intestinal surgery			0.302
No	55 (64.0%)	30 (73.2%)	—
Yes	31 (36.0%)	11 (26.8%)	—
History of perianal surgery			0.772
No	48 (55.8%)	24 (58.5%)	—
Yes	38 (44.2%)	17 (41.5%)	—
History of hormone therapy			**0.067**
Yes	29 (33.7%)	11 (26.8%)	—
No	49 (57.0%)	30 (73.2%)	—
Not sure	8 (9.3%)	0 (0.0%)	—
History of immunosuppressant therapy			0.768*
Yes	45 (52.3%)	21 (51.2%)	—
No	36 (41.9%)	19 (46.3%)	—
Not sure	5 (5.8%)	1 (2.4%)	—
History of biologic agent therapy			0.369
No	17 (19.8%)	11 (26.8%)	—
Yes	69 (80.2%)	30 (73.2%)	—
Reason of recent drug withdrawal^b^			
Unresponsive	48 (55.8%)	23 (56.1%)	0.976
Intolerant	8 (9.3%)	6 (14.6%)	0.370
Adverse effect	9 (10.5%)	4 (9.8%)	1.000*
Economic reason	8 (9.3%)	9 (22.0%)	**0.050**
Other	23 (26.7%)	11 (26.8%)	0.992

Self-rate health was still significantly different (*p* = 0.008), regardless of whether they had a low impact on everyday life. Other factors found to be associated with impact preference in the univariate analysis were a history of hormone therapy (*p* = 0.067) and economic reasons for recent drug withdrawal (*p* = 0.050).

^a^ *n* = 112.

b Multiple choice.

^#^Wilcoxon test.

*Fisher’s exact test.

**TABLE 9:** Multivariate logistic regression analysis of predictive factors for preference toward impact of UST administration on everyday life.

Variable	Or (95% CI)	*p*-Value
Male	0.78 (0.31–1.96)	0.600
Age at diagnosis	1.00 (0.93–1.07)	0.947
Marital status		
Single	Ref	—
Married	0.23 (0.06–0.95)	**0.042**
Divorced	3.71 (0.06–223)	0.531
Household income per capita (Yuan/month)		
≤5,000	Ref	—
5,001–10,000	4.39 (1.39–13.9)	**0.012**
10,001–20,000	1.58 (0.31–7.99)	0.582
≥20,001	26.1 (1.92–353)	**0.014**
Self-rated health		
Poor	Ref	—
Fair	12.10 (0.38–390)	0.160
Good	16.69 (1.57–177)	**0.020**
Very good	31.94 (2.94–347)	**0.004**
History of hormone therapy		
No	Ref	—
Yes	0.61 (0.23–1.61)	0.314
Not sure	ns	0.973
Reason of recent drug withdrawal (economic reason)	4.84 (1.35–17.4)	**0.016**
Note. ns, not significant.		

Patients with a positive self-rated health status (good, *p* = 0.020; very good, *p* = 0.008) were correlated with a higher possibility of UST preference with a low interference in everyday life.

## Discussion

The principal aim of this study was to identify the reasons and possible contributing factors for UST preference in the Chinese population with CD. To our knowledge, this is the first and most current multicenter cross-sectional study to investigate the preference for choosing UST and to identify the potential contributing factors for frequency and mode of administration in subgroup analyses among Chinese patients with CD.

This questionnaire-based study demonstrated that effectiveness, safety, frequency of administration, and mode of administration were the primary reasons for choosing UST. Patients who chose UST with a low frequency of administration and a convenient mode of administration were more likely to have a better self-rated health status. In China, most patients with CD chose UST for treatment due to steroid dependence, primary unresponsiveness to IFX, adalimumab, and immunosuppressants, or loss of response in long-term follow-up. IFX is the most common biological agent for the treatment of CD. Despite the high cost of UST, selecting UST also has various advantages compared with IFX, including efficacy and safety. Moreover, UST has a lower frequency of administration (three times in the first 6 months, thereafter once every 12 weeks) than IFX (three times in the first 6 weeks; thereafter, once every 8 weeks), which may be beneficial for patients in terms of saving time. In addition, after the first intravenous treatment of UST at the hospital, patients can subcutaneously inject UST by themselves at home for the second time, which is more convenient for patients who have less time for hospital admittance, such as international students, young people, and businessmen. The results of our study are similar to those of several recent Western studies (Constantinescu et al., 2009; Vavricka et al., 2012). Although the results from those studies were about IFX, all patients were more likely to select a subcutaneous injection strategy.

Patients reported that the major information source for UST information was from physicians, and nearly half of the patients reported shared decision making with respect to UST treatment. In this digital age, patients prefer to educate themselves and research on the benefits and risks of their therapy, and actively participate in the decision-making process of treatment (Guadagnoli and Ward, 1998; Siegel, 2012). Patients are more likely to be involved in the treatment of inflammatory bowel disease because of the uncertainty of the evidence regarding many clinical questions and the heterogeneity of the disease course (Siegel, 2012). Sharing decision making with patients is significant for improving clinical outcomes, resulting from a better adherence to the therapy. We believe that our results will facilitate Chinese patients with CD to make informative decisions.

This study had several strengths. First, patients were from 27 inflammatory bowel disease referral centers in different regions of China and represented a broad and reliable spectrum of the Chinese population. Second, this study was based on a well-designed questionnaire that contained detailed information about demographic and clinical characteristics at diagnosis and preference for UST from the perspectives of the patients. Third, we investigated possible demographic characteristics contributing to preference for UST in terms of frequency of administration, mode of administration, and interference with everyday life separately, which had not been analyzed in previous studies.

However, there are some limitations to the present study. First, generalization and extrapolation of the results in our study are questionable as genotypic and phenotypic differences exist in different regions of Asia. Multicenter studies targeting other Asian populations are needed in further work. Second, the factors for effectiveness and safety in UST preferences were not analyzed. Instead, the current study was the first to focus on the frequency and mode of administration. Finally, noncontinuity and intergenerational effects might have influenced the results owing to a cross-sectional study design. Further prospective studies may be the preferred choice to focus on the following treatment strategies after UST and associated factors.

## Conclusion

In conclusion, we found that the effectiveness, safety, and frequency of administration were the three main reasons patients chose UST. Patients who chose UST due to low frequency and administration convenience were more likely to have a better self-rated health status. Treatment choices should be discussed with patients as individual preferences are determined by diverse factors.

## Data Availability

The original contributions presented in the study are included in the article/Supplementary Material. Further inquiries can be directed to the corresponding author.
