# Supplementary material for: Reasons and Factors Contributing to Chinese Patients’ Preference for Ustekinumab in Crohn’s Disease: A Multicenter Cross-Sectional Study
Source: Front Pharmacol. 2021 Nov 22;12:736149. doi: 10.3389/fphar.2021.736149 (PMC8651007; doi:10.3389/fphar.2021.736149)
Supplement: Supplementary file 1 [file Table1.docx]

# Supplementary Table 1.The Questionnaire in Chinese patients with moderate to severe CD who underwent UST treatment

**《中国中重度CD患者乌司奴单抗治疗现况问卷调查》**

尊敬的克罗恩病患者:

您好！本次我们将以网络问卷的形式开展一项关于中国中重度CD患者使用乌司奴单抗治疗的横断面调查，开始前请您仔细阅读。

**一 问卷背景：**

克罗恩病（CD）是一种慢性迁延性疾病，有终生复发倾向，对患者的工作与生活造成了严重影响。乌司奴单抗在全球的多项临床研究显示对于中重度CD患者的诱导缓解及维持治疗有较好疗效。目前乌司奴单抗已在中国上市，并在国内多个地区应用。因此，开展此次问卷调查对于了解中国中重度CD患者乌司奴单抗治疗的现况具有极其重要的意义。

**二 问卷内容：**

（共46题，预计填写时间10分钟）

**1. 患者基本信息：**

姓名，联系方式，出生年月，性别，身高，体重，吸烟，饮酒，教育程度，婚姻状况，就业状态，平均月收入，家庭人均月收入，当前居住地，当前就诊医院，医保类型，医保所在地（本部分共计17题）；

**2. 患者疾病信息：**

发病日期，诊断日期，健康状态，疾病部位，疾病行为，家族史，胃肠道手术史，肛周手术史，既往治疗药物，最近一次药物中止原因（本部分共计25题）；

**3. 乌司奴单抗相关信息：**

本次可选择的药物，乌司奴单抗治疗CD的信息来源，选择乌司奴单抗的原因，选择乌司奴单抗的治疗决策方式（本部分共计4题）。

**三 注意事项：**

1. 问卷发放后，需在2周内完成并提交。

2. 调查期间您的信息将以调查编号数字而非您的姓名加以标识，任何调查组外成员均不能识别您身份的信息，如您的姓名、地址和电话等，除非事先获得您的许可。

3. 问卷调查组负责妥善保管您的资料信息，原则上在调查结束后销毁。

4. 您可以选择不参与本调查，您的任何医疗待遇与权益不会因此而受到影响。

5. 您的参与不会引起您正常医疗以外的花费。项目负责单位为非盈利机构，无任何商业行为，个人的参与不会因此获得任何经济利益。

**四 问卷详细内容：**

**1. 患者基本信息：**

姓名：___________

联系方式：__________

出生年月: ______年______月

性别：1.男 2.女

身高：______cm（0.1）

体重：______kg（0.1）

吸烟：1.从未吸烟 2.已戒烟 3.正在戒烟 4.仍在吸烟

饮酒：1.从未饮酒 2.饮酒少于1月1次 3.饮酒介于1周1次和1月1次之间 4.饮酒多于1周1次

教育程度：1.高中以下 2.高中 3.专科 4.本科 5.研究生及以上

婚姻状况：1.未婚 2.已婚 3.离异 4.丧偶

就业状态：1.学生 2.全职 3.兼职 4.待业 5.务农 6.退休 7.其他

平均月收入：________元

家庭人均月收入：________元

当前居住地：层次选项

当前就诊医院：_________________

医保类型：1.城镇职工医保 2.城镇居民医保 3.新农合 4.商业医保 5.自费

医保所在地：层次选项

**2. 患者疾病信息：**

发病日期：______年______月

诊断日期：______年______月

您认为自己现在的健康状态如何？1.很好   2.好   3.一般   4.差

与同龄人相比，您认为自己现在的健康状态如何？1.好于同龄人   2.差不多  3.更差   4.不知道

疾病部位：1.上消化道 2.小肠 3.结肠 4.不知道（可多选）

疾病行为：1.非狭窄非穿透型 2.狭窄型 3.穿透型 4.非狭窄非穿透型伴肛周病变 5.狭窄型伴肛周病变 6.穿透型伴肛周病变 7.不知道

克罗恩病家族史：无/有

您是否有过肠道部分切除、狭窄成形等胃肠道手术史：无/有

您是否有过肛瘘挂线、肛周脓肿切开引流等肛周手术史：无/有

既往治疗药物：

激素：开始时间______年______月 结束时间______年______月

免疫抑制剂：名称______ 开始时间_____年_____月 结束时间_____年_____月

英夫利昔单抗：开始时间______年______月 结束时间______年______月

阿达木单抗：开始时间______年______月 结束时间______年______月

维多珠单抗：开始时间______年______月 结束时间______年______月

最近一次药物中止原因：1.失应答（药物使用初期或使用一段时间后对症状控制不佳） 2.不耐受（使用药物期间出现身体不适等情况） 3.副作用 4.经济原因 5.其他

**3. 乌司奴单抗相关信息：**

本次可选择的药物：1.乌司奴单抗 2.英夫利昔单抗 3.阿达木单抗 4.维多珠单抗 5.激素 6.免疫抑制剂 7.其他_____（可多选）

乌司奴单抗治疗的信息来源：1.专科医生 2.家人、朋友、患友群 3.网络电视（电脑、手机、电视等） 4.纸质媒介（书籍、宣传册、杂志等）（可多选）

选择乌司奴单抗的原因：1.有效性 2.安全性 3.起效时间快 4.给药间隔长 5.给药方式方便 6.给药时长短 7.给药地点方便 8.给药者 9.对日常生活的干扰小 10.费用 11.其他_______（可多选）

选择乌司奴单抗的治疗决策方式：1.我和医生共同决定 2.在医生对不同方案解释后我自己作出决定 3.医生根据治疗作出决定

**The Questionnaire in Chinese patients with moderate to severe CD who underwent ustekinumab treatment**

Dear patients,

We will conduct a cross-sectional study in Chinese patients with moderate to severe CD who underwent ustekinumab (UST) treatment in the form of an online questionnaire, please read the following instructions before you start to answer.

**Backgrounds:**

Crohn’s disease (CD) is a chronic disorder of the gastrointestinal tract that requires life-long medical treatment, which might cause severe effects in work and daily life. Global clinical trials have revealed considerable effects of UST in the induction and maintain of remission for patients with moderate to severe CD. Currently, UST has been utilized among the Chinese population. Therefore, it is important to make this investigation to realize the current status of patients with moderate to severe CD who underwent ustekinumab (UST) treatment.

**Contents**(46 items，estimated time of 10 min):

**1. Basic information:**

Name, telephone number, birthday, gender, height, weight, smoking status, alcohol consumption, education, marital status, employment status, average monthly income, family per capita monthly income, current place of residence, current hospital visit, type of health insurance, location of medical insurance.

**2. Disease information:**

Date of onset, date of diagnosis, health status, disease location, disease behavior, family history, history of gastrointestinal surgery, history of perianal surgery, history of medications before survey, reason for the latest drug discontinuation.

**3. UST associated information:**

Optional drugs, information source, reasons for UST preference, strategy for UST drug choice

**Notes:**

1. You should complete and submit the questionnaire within 2 weeks.

2. Your information will be identified as the survey number rather than your name during the survey, and no members except the investigation group will be able to identify your information unless your permission is obtained.

3. The investigation group takes the responsibility for proper custody of your information and destroying it after the investigation has been completed.

4. You have the right whether to participate in this survey, your medical treatment and benefits will not be affected.

5. Your participation in this survey will not incur extra expenses other than your regular medical treatment. The project is initiated by non-profit organizations with no business practices, and you will not gain any individual financial benefits.

**Detailed information about the questionnaire:**

**1. Basic information:**

1.1 Name

1.2 Telephone number

1.3 Birthday

1.4 Gender: male/female

1.5 Height

1.6 Weight

1.7 Smoking status: non-smoker/former smoker/under the status of quitting smoking/current smoker

1.8 Alcohol consumption: never /drinking less than once a month/drinking between once a week and once a month/drinking more than once a week

1.9 Education: High school or under/college/university/ Graduate or above

1.10 Marital status: Single/Married/Divorced

1.11 Employment status: Student/Full-time/Part-time/Unemployed/Farmer/Retirement/Other

1.12 Average monthly income

1.13 Family per capita monthly income

1.14 Current place of residence

1.15 Current hospital visit

1.16 Type of health insurance: Medical insurance for urban workers/Medical insurance for urban residents/New agricultural insurance/Commercial medical insurance/Out-of-pocket expenses

1.17 Location of medical insurance

**2. Disease information:**

2.1 Date of onset

2.2 Date of diagnosis

2.3 Self-rated health status: Very good/Good/Fair/Poor

2.4 Disease location: Upper gastrointestinal disease/ileal/colonic/ileocolonic/unknown (multiple choice)

2.5 Disease behavior: Non-stricturing, non-penetrating/stricturing/penetrating/Non-stricturing, non-penetrating with perianal involvement/stricturing with perianal involvement /penetrating with perianal involvement/unknown

2.6 Family history: Yes/No

2.7 History of gastrointestinal surgery: Yes/No

2.8 History of perianal surgery: Yes/No

2.9 History of medications before survey:

Corticosteroids: Initiating time, ceasing time

Immunosuppressants: Initiating time, ceasing time

Infliximab: Initiating time, ceasing time

Adalimumab: Initiating time, ceasing time

Vedolizumab: Initiating time, ceasing time

2.10 Reasons of recent drug withdrawal: unresponsive (poor control of symptoms at the beginning of drug use or after a period of use)/Intolerant (physical discomfort during drug use) /Adverse effect/ Economic reasons/Other

**3. UST associated information:**

3.1 Optional drugs: Corticosteroids/Immunosuppressants/Infliximab/Adalimumab/Vedolizumab/UST/Others

3.2 Information source:

Physicians/Family/friends/patients association/Internet/Books/TV/Others

3.3 Reasons for UST preference:

Efficacy/Safety/Fast to response/Frequency of administration/Time of administration/Mode of administration/Place of administration/Selfcare/Interference with everyday life/Others

3.4 Strategy for UST drug choice: Decided by physicians and me/ Decided by myself after explanations from physicians/ Decided by physicians
