# Supplementary material for: Reasons and Factors Contributing to Chinese Patients’ Preference for Ustekinumab in Crohn’s Disease: A Multicenter Cross-Sectional Study
Source: Front Pharmacol. 2021 Nov 22;12:736149. doi: 10.3389/fphar.2021.736149 (PMC8651007; doi:10.3389/fphar.2021.736149)
Supplement: Supplementary file 2 [file Table2.docx]

**Supplementary Table 2. Comparison of strategy on UST drug choose between patients from Hubei province and other provinces**

| Strategy on drug choose | Patients from Hubei province n(%) | Patients from other province n(%) | *P*-value |
| --- | --- | --- | --- |
| Decided by physicians and me | 9（39%） | 41（46%） | 0.55 |
| Decided by myself after explanations from physicians | 12（52%） | 32（36%） | 0.16 |
| Decided by physicians | 2（9%） | 16（18%） | 0.28 |
