# Supplementary material for: Reasons and Factors Contributing to Chinese Patients’ Preference for Ustekinumab in Crohn’s Disease: A Multicenter Cross-Sectional Study
Source: Front Pharmacol. 2021 Nov 22;12:736149. doi: 10.3389/fphar.2021.736149 (PMC8651007; doi:10.3389/fphar.2021.736149)
Supplement: Supplementary file 3 [file Table3.docx]

**Supplementary Table 3. Comparison of** **reasons for UST preference between patients from Hubei province and other provinces**

| Reasons for UST preference | Patients from Hubei province n(%) | Patients from other province n(%) | *P*-value |
| --- | --- | --- | --- |
| Efficacy | 18(78.3%) | 66(74.2%) | 0.69 |
| Safety | 12(52.2%) | 60(67.4%) | 0.17 |
| Fast to response | 6(26.1%) | 27(30.3%) | 0.69 |
| Frequency of administration | 5(21.7%) | 4(4.5%) | 0.007 |
| Time of administration | 1(4.3%) | 19(21.3%) | 0.06 |
| Mode of administration | 7(30.4%) | 35(39.3%) | 0.43 |
| Place of administration | 3(13.0%) | 7(7.9%) | 0.44 |
| Selfcare | 4(17.4%) | 10(11.2%) | 0.43 |
| Interference with everyday life | 7(30.4%) | 30(33.7%) | 0.77 |
| Others | 2(8.7%) | 5(5.6%) | 0.59 |
